# Mental Health and Age-Related Differences in Community During the COVID-19 Pandemic: A Cross-Sectional Study from Southeastern Türkiye

**DOI:** 10.3390/medicina61101840

**Published:** 2025-10-14

**Authors:** Pakize Gamze Erten Bucaktepe, Vasfiye Demir Pervane, Ömer Göcen, Sercan Bulut Çelik, Fatima Çelik, Öznur Uysal Batmaz, Ahmet Yılmaz, Tahsin Çelepkolu, Kürşat Altınbaş

**Affiliations:** 1 Department of Family Medicine, Medical Faculty, Dicle University, Diyarbakır 21280, Türkiye; vasfiyyedemir@hotmail.com (V.D.P.); ahmet.yilmaz@dicle.edu.tr (A.Y.); tcelepkolu@gmail.com (T.Ç.); 2 Kayapınar Talaytepe Family Health Center, Provincial Health Directorate, Diyarbakır 21070, Türkiye; omergocen65@gmail.com; 3 Batman GAP Family Health Center, Provincial Health Directorate, Batman 72070, Türkiye; bulutum07@yahoo.com; 4 Provincial Health Directorate, Public Health Directorate Services Training Unit, Batman 72060, Türkiye; celik_fatima@hotmail.com; 5 Department of Internal Medicine, University of Health Sciences Gazi Yaşargil Training and Research Hospital, Diyarbakır 21090, Türkiye; oznrbtmz@gmail.com; 6 Department of Psychiatry, Atlas University, İstanbul 34403, Türkiye; kursataltinbas@gmail.com

**Keywords:** anxiety, depression, stress, coping flexibility, social support, age groups, COVID-19 pandemic

## Abstract

*Background and Objectives:* The COVID-19 pandemic has caused profound disruptions in socioeconomic, and health domains, with significant implications for mental well-being. The aim of this study was to evaluate the impact of the pandemic on stress, anxiety, and depression, alongside perceived social support, coping flexibility and related factors, and to examine how these issues vary across different age groups. *Materials and Methods:* A cross-sectional analytical study was conducted in Türkiye between August and December 2020. Data were collected through an online questionnaire including sociodemographic characteristics, pandemic-related concerns, and validated scales: Hospital Anxiety and Depression Scale (HADS), Perceived Stress Scale (PSS), Coping Flexibility Scale (CFS), and Multidimensional Scale of Perceived Social Support (MSPSS). Statistical analyses included descriptive and comparative tests, correlation analysis, multiple linear regression models, and correspondence analysis. *Results:* Among 1699 participants, 58.0% were female; 24.5% and 42.1% reported anxiety and depressive symptoms above thresholds, respectively. Younger age correlated negatively with stress, anxiety, and depression scores (*p* < 0.001). Feelings of loneliness, loss of control, ostracism, and sleep or concentration problems were positively associated with anxiety, depression, and stress, but negatively associated with coping flexibility and social support (*p* < 0.001). The 15–20 age group had the highest anxiety and depression levels and the lowest social support; the 15–30 group showed the highest stress, while the 61–75 group exhibited the lowest coping flexibility. Regression models explained 62.7% of anxiety and 56.6% of depressive symptom variances. Major predictors of anxiety included depressive symptoms, stress, and fear of dying from COVID-19, while depressive symptoms were predicted by age, stress, coping flexibility, social support, and anxiety. *Conclusions:* The findings highlight the considerable psychological burden and distinct vulnerabilities among age groups. Mental health interventions should be tailored according to age, emphasising the enhancement of social support and coping flexibility to strengthen resilience in future pandemics.

## 1. Introduction

The coronavirus 2019 (COVID-19) pandemic has profoundly disrupted individual and collective well-being, posing unprecedented challenges to global public health [1,2]. Although COVID-19 directly affects infected individuals, its psychosocial consequences jeopardise the mental health of the entire community [3]. Beyond its physical health impact, the pandemic triggered widespread psychological distress, with increases in depression, anxiety, and stress linked to fear, stigma, social isolation, and economic disruption [4,5,6]. A substantial body of research that examined the mental health impacts of the pandemic, have revealed substantial heterogeneity across demographic groups, suggesting that psychological vulnerability and resilience may vary depending on age, gender, and social context and can persist long after their outbreak [6,7,8,9,10].

Studies also report that working status/conditions, physical activity levels and health consequences may impact one’s risk of experiencing mental health problems within this context [8,11]. For example, it has been estimated that future pandemics might be associated with increased mental health vulnerability in females, youth, healthcare workers, older adults, and people with chronic conditions and financial challenges [5,12,13]. In addition, feelings of loneliness, fear of death/losing control over one’s life, and concentration and sleep problems may act as stressors that further compound such issues [14]. However, the ways in which individuals cope with these stressors, the dynamics of their social relationships, and how they maintain psychological resilience may vary depending on personal, cultural, and environmental factors [15].

In such challenging periods, individuals’ ability to cope effectively and their access to social support become vital determinants of mental well-being [16]. Coping flexibility—the capacity to adaptively modify coping strategies in response to stressful or traumatic circumstances—has been identified as a key resilience factor that helps maintain psychological stability under pressure [17,18]. For example, in Italy, two studies highlighting the importance of internal emotion regulation processes in the Italian context suggested that coping flexibility and emotional regulation might play a particularly salient role during pandemic stress and justify including coping flexibility as a key variable [19,20]. Likewise, social support serves as a protective mechanism by providing emotional, cognitive, and financial resources that buffer the adverse effects of stress and anxiety [21,22]. However, social support does not solely refer to physical proximity or in-person interactions. Yet, during infectious disease outbreaks, the nature of social support becomes more complex; physical proximity may represent a potential health risk, while virtual and emotional forms of support gain prominence as alternative protective mechanisms including digital communication and community networks [23]. Thus, the quality and perceived availability of supportive relationships—rather than mere physical presence—are key determinants of individuals’ psychological resilience and ability to cope effectively during such crises [22]. Additionally, the influence of these protective factors appears to differ across the lifespan [23]. Emerging evidence indicates that younger individuals have been disproportionately affected by the psychosocial consequences of the pandemic, often reporting elevated stress, anxiety, and depressive symptoms compared to older adults [23,24]. These patterns have been attributed to social restrictions, disruptions in education and employment, and reduced access to peer support networks [23]. Conversely, older adults—while potentially more resilient due to life experience—have faced increased risks of loneliness and depressive symptoms, exacerbated by health vulnerabilities and limited social interaction [24]. However, the mechanisms underlying these age-related differences, particularly the roles of protective factors such as coping flexibility and social support, remain insufficiently explored. Understanding how this resilience factors operate across different age groups is therefore essential for designing targeted interventions and promoting mental health during prolonged crises.

The pandemic has provided a unique opportunity to examine how extraordinary circumstances influence psychological well-being across the lifespan. Numerous studies conducted both globally and in Türkiye have focused on specific age groups—such as adolescents, young adults, middle-aged adults, or older adults—highlighting distinct vulnerabilities and coping patterns within each cohort [17,25,26,27]. For instance, some studies have explored mental health outcomes among older and middle-aged populations [28], some compared groups aged over 60 years [12], or categorised adults over 18 into multiple age strata [23,24]. Despite these efforts, the literature still lacks studies that evaluate both adolescents and older adults simultaneously and categorise participants into decade-based age groups, offering a more objective and comprehensive understanding of age-related differences. Moreover, findings regarding the mental health effects of COVID-19 across age groups remain inconsistent, suggesting that sociopolitical, geographic, and cultural factors may play a role in shaping individuals’ perceptions and emotional responses to crises [29]. Importantly, mental health does not appear to vary linearly with age; some evidence suggests that age may exhibit a curvilinear relationship with psychological outcomes during crises such as pandemics, reflecting nonlinear patterns of vulnerability and resilience [30]. Therefore, examining individuals across age decades provides a more nuanced and balanced perspective on how stress, anxiety, depression, and protective factors—such as coping flexibility and social support—manifest at different stages of life.

Addressing these gaps, the present study aims to investigate the interrelations among perceived stress, anxiety, depression, coping flexibility, and perceived social support during the COVID-19 pandemic, while identifying sociodemographic and COVID-related determinants that influence these outcomes and to evaluate the variations and distribution trends of mental health issues depending on age. By analysing age-specific variations in a large community sample from Türkiye, this study contributes to the literature by filling a critical gap in understanding how mental health risk and resilience differ across decades of life. The findings will provide empirical evidence to support the development of targeted, age-sensitive, community-oriented public health strategies and psychological interventions for future global crises. Building on this rationale, our hypothesises are:

**Hypothesis 1 (H1).**  *Participants would exhibit higher levels of anxiety, depressive symptoms and perceived stress during the COVID-19 pandemic.*

**Hypothesis 2 (H2).** 
*Sociodemographic factors (e.g., age, gender, employment status), COVID-19-related characteristics (e.g., fear of infection, loneliness), coping flexibility and perceived social support would significantly predict anxiety and depression.*

**Hypothesis 3 (H3).** 
*Younger and older age groups would report higher levels of stress, anxiety, and depression, but lower levels of coping flexibility and perceived social support compared to middle aged groups.*

## 2. Materials and Methods

### 2.1. Design

This cross-sectional analytical study was conducted in a Southeastern province of Türkiye between August and December 2020. All methods were performed in accordance with the STROBE checklist to improve the quality of the study [31]. Information about the study, an informed consent form consisting of the purpose, procedures and requirements of the study, a data form including sociodemographic and COVID-related features and problems, and the measurement scales were uploaded to Google Forms. Taking into consideration the need to preserve social distancing, the questionnaire was delivered online through Google Forms to be completed via smartphone, the URL for which was sent using WhatsApp, the most preferred social media platform in Türkiye. When the survey items were created in Google Forms, they were set up so that respondents cannot proceed to the next question without answering the previous one. As the questionnaire could not progress without an answer, there was no missing data.

### 2.2. Sample Size and Participants

A total of 1699 participants (aged 15–75) were reached online through the convenience sampling method. This method was preferred because COVID-19 lockdown measures were in place during the study period and people were afraid of direct contact and avoided it. After having been taken the ethical approval, participants were invited online using family medicine system records of family healthcare centres where the researchers worked with the permission of the Provincial Health Directorate and then snowball dissemination method was used. Participation was voluntary, and no explicit inclusion or exclusion criteria were applied except from adults aged 15–75 with internet access. Due to low literacy and internet usage rates among the elderly in our country, the representation of those aged 61 and over in the study was low. For adolescent participants aged 15–18, researchers working within the family medicine system first contacted their parents in accordance with ethical guidelines. After obtaining consent for their children’s participation, the link was sent via the parents to the adolescents. Post hoc power analysis using G*Power 3.1.9.7 indicated that the sample size was sufficient.

### 2.3. Ethical Considerations and Consent Statement

Approval for the study was granted by the Dicle University Faculty of Medicine Non-Interventional Clinical Research Ethics Committee (2020/199). Permission was also obtained from the Provincial Directorate of Health. The study was conducted following the Helsinki Declaration ethical guidelines and the ethical principles of ‘Informed Consent, Confidentiality and Protection of Privacy and Respect for Autonomy’. The participants were informed that their participation was voluntary, they could withdraw at any point, and their responses would remain confidential and anonymous. The questionnaire was designed to be completed after information was read and consent was given.

### 2.4. Data Collection Tools

Questions about sociodemographic characteristics (gender, age, marital status, education, employment, chronic disease, chronic patient at home, no of household members), COVID-related descriptive features as easy access to Personal Protective Equipment (PPE), having been quarantined, need for psychologic support were asked to respond with either no or yes.

Participants were also directly asked to answer the questions about six potential problems related to COVID-19 scored as 1 = no, 2 = occasionally, 3 = sometimes, 4 = often, and 5 = always, as follows: “Are you feeling lonely during the COVID-19 pandemic?”, “Do you feel distant from other people during the COVID-19 pandemic?”, “During the COVID-19 pandemic, do you feel as though you have lost control of your life?”, “Do you feel ostracised during the COVID-19 pandemic?”, “Are you experiencing sleep problems (such as difficulty falling asleep, sleeping too much, or sleeping too little) during the COVID-19 pandemic?” and “Are you finding it difficult to concentrate during the COVID-19 pandemic?”.

Numeric Rating Scales (NRSs) graded from 0 (I am not afraid) to 10 (I am very much afraid) were used to determine the fear of contracting the virus and dying from COVID-19. The NRS was reported to be valid, reliable and sensitive by Becker et al. [32]. The responses were taken as continuous variables in the evaluation, with higher scores indicating greater problems.

### 2.5. The Hospital Anxiety Depression Scale (HADS)

The HADS was developed by Zigmond and Snaith to determine an individual’s risk of anxiety and depression [33] and translated into Turkish by Aydemir [34]. It consists of 14 items on a 4-point Likert-type scale, 7 of which (odd numbers) measure anxiety and 7 of which (even numbers) measure depression. An example item of anxiety is “I feel tense and wound up” and an example item for depression is “I still enjoy the things I used to enjoy”. The cutoff points are 10/11 for the anxiety subscale (HADS-A) and 7/8 for the depression subscale (HADS-D), where higher scores indicate a higher risk. For this study, cutoff values of 11 for HADS-A and 8 for HADS-D were utilised, and the internal consistency coefficient (Cronbach α) was determined as 0.847 for anxiety and 0.782 for depression.

### 2.6. The Coping Flexibility Scale (CFS)

The CFS was developed by Kato to measure a person’s ability to try new methods of coping with stressful life events, and it was translated into Turkish by Akın et al. [35,36]. It utilises a four-point Likert-type scale featuring 10 questions, scored from 0 (not appropriate) to 3 (quite appropriate), where higher scores indicate greater coping flexibility. An example item is “If I feel I cannot cope with stress, I change my coping method”. The Cronbach α value of the sample was determined as 0.827.

### 2.7. The Perceived Stress Scale (PSS)

The PSS was developed by Cohen et al. to measure an individual’s perceived stress within the last month and translated into Turkish by Eskin et al. [37,38]. It is a 5-point Likert-type scale with 14 items, scored from 0 (never) to 4 (too often), where higher scores indicate greater stress perception. An example item is “In the last month, how often have you been upset because of something that happened unexpectedly?”. The Cronbach α value of this study was calculated as 0.858.

### 2.8. Multidimensional Perceived Social Support Scale (MPSSS)

The MPSSS is a 12-item scale that subjectively evaluates the adequacy of social support received from three different sources: family, friends, and significant others. The scale was developed by Zimet et al. and translated into Turkish by Eker and Arkar [39,40], composed of a 7-point Likert-type scale scored from 1 (absolutely no) to 7 (absolutely yes), where higher scores indicate greater perceived social support. An example item is “There is a special person who is around when I am in need”. The Cronbach α value of this sample was found to be 0.925.

The completed questionnaires were transferred from Google Forms to Microsoft Excel programme; after checking, the data were uploaded to IBM SPSS v. 27.0 for analysis.

### 2.9. Data Analysis

Data obtained in the study were analysed statistically using IBM SPSS v. 27.0 (IBM Corporation, Armonk, NY, USA). Conformity of the data to normal distribution was assessed based on skewness and kurtosis with positive results. Continuous variables are presented as mean ± standard deviation values and categorical variables are presented as numbers and percentages. Student’s *t* Test was used to compare differences between two independent means. One-Way or Welch ANOVA tests were used to compare more than two groups according to the Levene Homogeneity of Variances Test; when a significant result was found, comparisons of subgroups were made via pairwise post hoc Tukey’s HSD tests or Tamhane’s T2 Tests depending on appropriateness. The magnitudes of differences were assessed using Cohen’s d for Student’s *t* Test and using eta squared (η^2^) for ANOVA tests. All scale scores were standardised to examine how they change with age, and the ages of the participants were divided into six groups. We separately calculated the mean values of standardised z scores according to age groups, which were then shown graphically. To determine relationships between numerical data, Pearson correlation analysis was used. Multivariate Linear Regression Analysis was applied to the variables with significant correlations to determine the predictors of anxiety and depressive symptoms and enter method was used. Multicollinearity was assessed using Tolerance and Variance Inflation Factor tests, and no problems were identified. The normality, linearity, and homoscedasticity of residuals were also considered. Correspondence analysis was used to determine the coping flexibility, perceived stress, perceived social support, and anxiety and depressive symptom status of different age groups of participants as the cumulative contribution of the first two factors to the variance was 98.5%, greater than the recommended value of 75%. In the correspondence analysis, the HADS-A and HADS-D subscales were analysed according to their cutoff values, while CFS, PSS, and MDPSS scores without cutoff values were analysed after division into two groups according to their mean values (i.e., below and above the mean). The internal consistency of the scales was determined for this sample using the Cronbach α indicator. Hypotheses were two-sided, and *p* < 0.05 was accepted as statistically significant.

## 3. Results

Of the 1699 participants, 986 (58.0%) were female. Their mean age was 34.1 ± 13.1 (15–75) years; 417 (24.5%) had anxiety and 716 (42.1%) had depressive symptoms above the threshold levels. The sociodemographic and COVID-19-related features of the participants are shown in Table 1.

Table 2 shows the mean values of the scale scores, COVID-related problems and fears, and their correlations with scale scores. It was determined that age was inversely correlated with PSS, HADS-A and HADS-D scores (all *p* values < 0.001), directly correlated with MPSSS score (*p* < 0.001), and not correlated with CFS score (*p* = 0.173). Feeling lonely, feeling distant from others, the feeling of losing control over one’s life, feeling ostracised, and sleep and concentration problems were significantly and positively correlated with HADS-A, HADS-D, and PSS scores and significantly and negatively correlated with CFS and MPSSS scores (all *p* values < 0.001). Fear of contracting and dying from COVID-19 was found to be significantly and positively correlated with PSS (*p* values < 0.001), MPSSS (*p* = 0.047 and *p* = 0.019, respectively), HADS-A (*p* values < 0.001), and HADS-D (*p* values < 0.001) scores but negatively correlated with CFS scores (*p* < 0.001 and *p* = 0.003, respectively). PSS, HADS-A, and HADS-D scores were positively and significantly correlated with each other and negatively and significantly correlated with CFS and MPSSS scores (all *p* values < 0.001). CFS and MPSSS scores were significantly and positively correlated with each other (*p* < 0.001).

A comparison between scale scores and sociodemographic characteristics is presented in Table 3. The stress, anxiety, and depressive symptom scores of women (all *p* values < 0.001), single people (*p* < 0.001, *p* = 0.001, and *p* = 0.005, respectively), students (all *p* values < 0.001), those with chronic patients at home (all *p* values < 0.001), those who could not access PPE easily (*p* = 0.008, *p* < 0.001, and *p* < 0.001, respectively), those who had been in quarantine (*p* < 0.001, *p* < 0.001, and *p* = 0.002, respectively), and those who felt the need for psychological support (all *p* values < 0.001) were found to be significantly higher. The coping flexibility score was found to be significantly higher in those who were divorced or widowed (*p* = 0.031), in those with university education or higher (*p* = 0.002), and in those who had easy access to PPE (*p* < 0.001), while it was found to be significantly lower in students (*p* = 0.002), in those with chronic patients at home (*p* = 0.003), and in those who needed psychological support (*p* < 0.001). The perceived social support levels of married people (*p* < 0.001), those who had university or higher education levels (*p* = 0.019), and those who had easy access to PPE (*p* < 0.001) were significantly higher, while the perceived social support levels of students (*p* < 0.001), those with chronic patients at home (*p* < 0.001), those who were quarantined (*p* = 0.002), and those who needed psychological support (*p* < 0.001) were significantly lower. In these analyses, all effect sizes were calculated as being small except for PSS, HADS-A and HADS-D scores regarding the need for psychological support (with large effect sizes of −0.815, −1.101, and −0.933, respectively).

In the presence of anxiety symptoms, PSS and HADS-D scores were found to be significantly higher with large effect sizes (−1.090 and −1.350, respectively). On the other hand, CFS (medium effect size: 0.660) and MPSSS (small effect size: 0.411) scores were found to be significantly lower (all *p* values < 0.001). Similarly, it was determined that PSS and HADS-D scores were significantly higher with large effect sizes (−1.043 and −1.340, respectively), whereas CFS and MPSSS scores (medium effect sizes: 0.664 and 0.569, respectively) were significantly lower in the presence of depressive symptoms (all *p* values < 0.001).

In the comparison of scale scores and age groups, the post hoc analysis showed that anxiety and depressive symptom levels were significantly higher in the 15–20 age group than in the 41–50 (*p* = 0.030 and *p* = 0.031, respectively) and 51–60 (*p* = 0.004 and *p* = 0.005, respectively) age groups, as well as being higher in the 21–30 age group than in the 51–60 age group (*p* = 0.014 and *p* = 0.012, respectively). The CFS score of the 51–60 age group was higher than that of the 61–75 age group (*p* = 0.021). The PSS scores of the 15–20 and 21–30 age groups were significantly higher than those of the other age groups (all *p* values < 0.01). The MPSSS score level of the 15–20 age group was significantly lower than those of the 21–30, 31–40, 41–50, and 51–60 age groups (all *p* values < 0.01) (Table 3).

**Figure 1:**
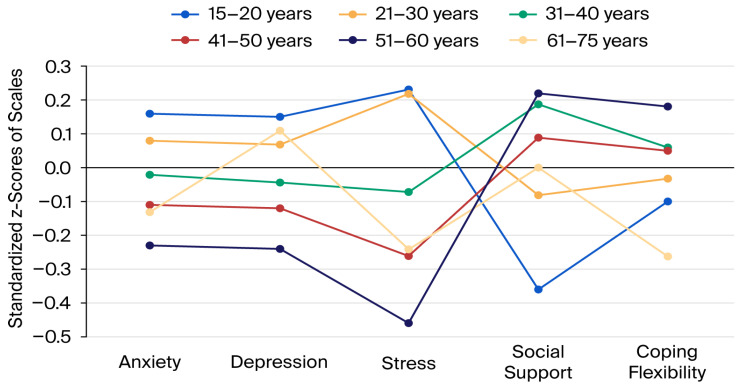
Relationships between age groups and scale scores of the participants (the 0.0 level indicated in black represents the average scale scores and z-scores above 0 indicate higher-than-average symptom levels).

The relationships between the standardised scale scores and age groups are shown in Figure 1. In the 15–20 and 21–30 age groups, the average scores for anxiety, depression and perceived stress were above the mean, while those for perceived social support and coping flexibility were below the mean. In contrast, in the 31–40, 41–50, and 51–60 age groups, the average scores for anxiety, depression and perceived stress were below the mean, while those for perceived social support and coping flexibility were above the mean. In the 61–75 age group, the only score above the average was that for depression.

Linear regression analyses were performed to determine the effect of age, COVID-related problems and fears, and scale scores on anxiety and depressive symptoms (Table 4). The models explained 62.7% (F:205.025, *p* < 0.001) and 56.6% (F:159.412, *p* < 0.001) of the anxiety and depressive symptom variances, respectively. Significant factors related to anxiety symptoms were the number of people living at home (β = 0.040; *p* = 0.011), the feeling of losing control over one’s life (β = 0.045; *p* = 0.037), feeling ostracised (β = 0.073; *p* < 0.001), sleep problems (β = 0.059; *p* = 0.004), concentration problems (β = 0.079; *p* < 0.001), fear of contracting COVID-19 (β = 0.097; *p* < 0.001), fear of dying from COVID-19 (β = 0.126; *p* < 0.001), coping flexibility (β = −0.050; *p* = 0.003), stress (β = 0.197; *p* < 0.001), and depressive symptoms (β = 0.399; *p* < 0.001). Significant factors related to depressive symptoms were age (β = 0.045; *p* = 0.009), coping flexibility (β = −0.092; *p* < 0.001), stress (β = 0.187; *p* < 0.001), social support (β = −0.087; *p* < 0.001), and anxiety symptoms (β = 0.464; *p* < 0.001).

**Figure 2:**
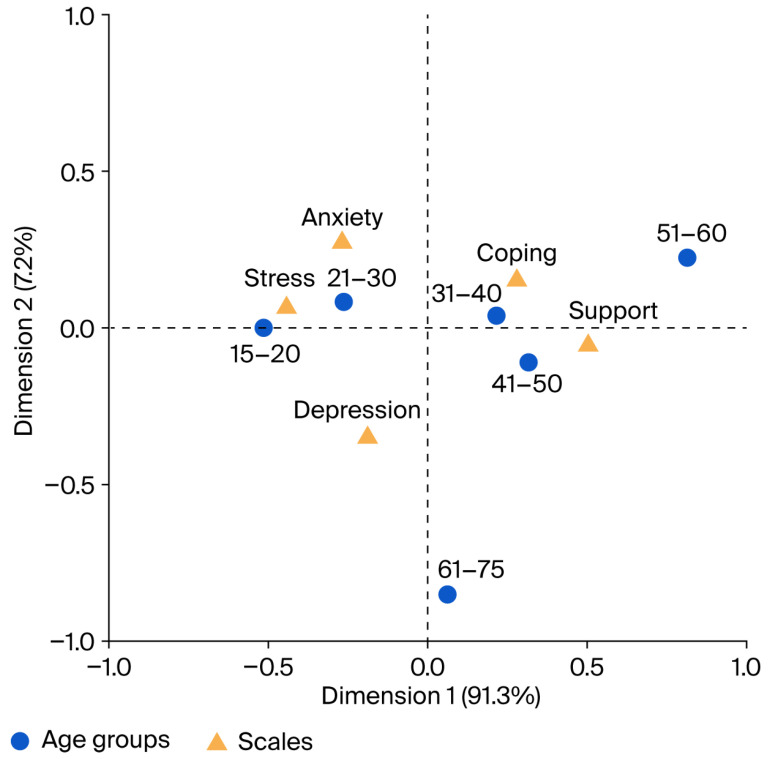
Factor loading chart from correspondence analysis for age groups and scales.

Figure 2 illustrates a plot of the corresponding factor loadings, showing that the 15–20 and 21–30 age groups may be at a higher risk for anxiety and stress in addition to a slightly lower risk of depression. The 31–40 and 41–50 age groups exhibit stronger associations with social support and coping flexibility, an observation also found for the 51–60 age group. Although those aged 61–75 do not exhibit close associations with any of the measured factors, this group’s greatest risk is depression.

In summary, depression was particularly prevalent among participants (42.1%). Younger age groups displayed higher anxiety, depression and stress, and poorer social support and coping flexibility. Prominent findings in those over 61 years of age were increased depressive symptoms and lower coping flexibility. COVID-19-related fears, stress and coping flexibility were found to be the major mental health risk factors.

## 4. Discussion

This study revealed the mental health burden imposed by pandemics on communities, reflected in serious rates of anxiety (24.5%) and depressive symptoms (42.1%). In a systematic review evaluating the pandemic data of eight countries, including Türkiye, anxiety and depressive symptoms were reported at rates of 6.3–50.9% and 14.6–48.3%, respectively, with societies being highly prone to anxiety, depression and stress as a whole [3]. In another study conducted in Türkiye during the same period, anxiety and depression rates were reported as 45.1% and 23.6%, respectively [41]. According to the results of the 2016 and 2019 Turkish health surveys, the adult population’s point of prevalence of depressive symptoms stated as to be 4.7% ± 0.24 in males and 8.0% ± 0.19 in females, with a population total of 6.3% ± 0.21 before the pandemic [42]. Additionally, a meta-analysis conducted during the pandemic, Turkey was reported to be among the countries with higher-than-pooled prevalence in respect of anxiety [43]. This study showed that age was inversely correlated with PSS, HADS-A and HADS-D scores, directly correlated with MPSSS score, and not correlated with CFS score. Feeling lonely, feeling distant from others, the feeling of losing control over life, feeling ostracised, and sleep and concentration problems were positively correlated with HADS-A, HADS-D, and PSS scores and negatively correlated with CFS and MPSSS scores. Fear of contracting and fear of dying from COVID-19 were found to be positively correlated with PSS, MPSSS, HADS-A, and HADS-D scores but negatively correlated with CFS score. PSS, HADS-A, and HADS-D scores were positively correlated with each other and negatively correlated with CFS and MPSSS scores. CFS and MPSSS scores were positively correlated with each other. The publications show correlations between stress, anxiety, depression, fears related to COVID-19, and loneliness [13,44,45,46]. A study conducted in Austria during the first wave of the pandemic reported inverse correlations between social support and depression and anxiety [47]. A study from Israel also showed that depression and anxiety decrease with age and increase with loneliness, exhibiting a positive correlation with each other [48]. A study conducted in China at the beginning of the pandemic also found that effective coping and social support were inversely proportional to psychological problems, and that this could comprise a basis for psychological interventions [49]. Interestingly, a counterintuitive finding emerged, with higher COVID-19-related fears correlating with greater perceived social support. This may reflect that individuals experiencing heightened fear actively seek, or become more aware of, available supportive resources. Alternatively, reporting biases, such as social desirability or overestimation of support during periods of elevated anxiety, may have contributed to this association. Given the cross-sectional design, causal relationships cannot be inferred, and unmeasured factors such as personality traits or prior mental health status may also play a role. Future longitudinal studies are warranted to clarify the directionality and underlying mechanisms of this relationship and to better understand how perceived support interacts with fear in pandemic contexts.

Female gender, younger age, being single, being a student, having a chronic patient at home, problems in accessing PPE, having been quarantined, and feeling the need for psychological support were associated with anxiety, depression and stress. Similarly, a meta-analysis in the Saudi population identified female gender, youth, being single, low education and income, unemployment, student status, fear, quarantine, and social restrictions as risk factors [50]. Women were particularly at risk for mental illness during the pandemic [1,3], likely due to gender inequalities that increase stress because of job characteristics, childcare responsibilities, and higher exposure risk to intimate partner violence [1].

It was found that the 51–60 age group, divorcees/widows, those with university education or higher, employed people, and those with easy access to PPE showed greater coping flexibility, while those with chronic patients at home, who need psychological support, or who have anxiety and depressive symptoms above the threshold value experience greater difficulty in coping flexibly. Inevitably, workforces dominated by individuals with low education levels were affected worse by the pandemic. Additionally, the presence of chronic diseases worsening the prognosis of COVID-19 would make coping with the pandemic more difficult. On the other hand, the life experiences of divorced and widowed people could result in psychological growth and coping flexibility. A study conducted in Peru at the beginning of the pandemic discussed the association between psychological distress and poor coping responses, emphasising that health policies focused on developing effective coping strategies for individuals during pandemics could play a role in reducing psychological problems [51].

Social support was perceived to be higher in those who were married, had a university education or higher, had easy access to PPE, or had anxiety and depressive symptoms below the threshold value and lower in the 15–20 and 21–30 age groups, students, those with chronic patients at home, those who were quarantined, and those who needed psychological support. In a longitudinal study investigating the effects of the pandemic in Poland, the importance of social support and coping efficacy was emphasised, with their quantities related to distress and their qualities related to mental health [52]. Similarly, in a multicentre study conducted on college students in China during the early stages of the pandemic, which examined the effects of social support and coping styles on mental health, it was found that coping style and perceived social support were significantly associated with depression, anxiety, and stress, and that effective coping style and social support were protective factors for mental health [16]. Community-based programmes that strengthen social connectedness and promote coping flexibility could further mitigate stress and emotional distress. Policymakers should also integrate mental health components into pandemic preparedness and response plans, ensuring that psychosocial support, education, and outreach are distributed equitably across age groups. Additionally, individuals reporting a need for psychological support should be prioritised and referred to appropriate services.

This study showed high levels of stress, anxiety and depression and low levels of coping flexibility and perceived social support in the 15–20 and 21–30 age groups. Our findings are in parallel with many studies [8,44,50], a situation that has been attributed to the lifestyle characteristics of young people due to the fact that they mostly live alone and lack an income-generating job or work in jobs with a high probability of encountering the virus [53]. Additionally, the majority of these individuals are students lacking life experiences that provide opportunities for psychological growth, leading to a need for greater support [54]. Moreover, it was inevitable that young people would be more affected by the uncertainties created by the pandemic due to preexisting concerns regarding the future socioeconomic conditions in Türkiye. The high prevalence of anxiety and depressive symptoms, particularly among younger individuals, underscores the need for age-specific preventive and therapeutic strategies.

It is noteworthy that depression was disproportionately high compared with stress and anxiety levels in the 61–75 age group, which—to some extent—might be related to conditions independent of the pandemic, such as ongoing loneliness and chronic diseases. This is further supported by our results regarding lower perceived social support and coping flexibility in the elderly. Most elderly people are retired, some are widowed, and some have chronic illnesses that can affect their daily lives and social relationships, so their greatest social support comes from family and friends. Therefore, it is to be expected that the social restrictions experienced during the pandemic would have a greater impact on this population [12]. Given the health and social problems they face, coupled with their susceptibility to complications caused by COVID-19 and higher mortality rates, it is only natural that they are unable to cope flexibly. In addition, there exists a significant but intricate relationship between anxiety, depression, stress, social support and loneliness in the elderly [45]. In older populations, where coping flexibility was found to be lower, interventions focusing on resilience-building, cognitive engagement, and social inclusion are recommended. Another finding that warrants attention is the relatively lower levels of anxiety and stress observed in older adults. According to the Socioemotional Selectivity Theory (SST), as people grow older, they become aware of how much life is worth living and tend to spend their remaining time in a more socially and emotionally fulfilling manner [55]. Furthermore, SST, which argues that increasing life experiences with age teach people to appreciate what they have, suggests that this may enable older adults to express their emotions more clearly and manage their anxiety and stress more efficiently [55]. Moreover, Cartensen et al., in their study during the pandemic, reported that such positive emotional regulation could continue even under the threat posed by COVID-19 [56].

The linear regression analysis results validated the proposed models, explaining a considerable amount of variance in anxiety (62.7%) and depression (56.6%) scores. The number of people living at home, the feeling of losing control over life, feeling ostracised, sleep problems, concentration problems, fear of contracting COVID-19, fear of dying from COVID-19, stress, and depressive symptoms were associated with higher anxiety scores, whereas coping flexibility was associated with lower anxiety scores. Moreover, age, perceived stress, and anxiety symptoms had positive effects on depression, whereas coping flexibility and perceived social support had negative effects. It has been reported that fears, loneliness, and stress related to COVID-19 play important roles in the development of depression and anxiety, and that effective coping and social support are protective factors for mental health [13,16]. Studies show that loss of control, concentration and sleep problems, and fear and stress caused by COVID-19 also negatively affect mental health [44,57]. Our linear regression analysis did not reveal any association between depression and fears or situations arising during the COVID-19 pandemic, such as loneliness and sleep problems—a noteworthy finding. However, this result has been attributed to the cross-sectional design of the study, as making predictions about cause-and-effect relationships is only possible with longitudinal studies. For example, in a study examining the effect of loneliness on depression during two separate pandemic waves in China, it was found that loneliness during wave 1 was associated with depression during wave 2 [58]. Mental health services should prioritise the early identification of at-risk groups—especially young adults, women, and individuals experiencing loneliness, loss of control, or social isolation—and provide accessible, youth-friendly, and digitally delivered psychological support. Incorporating explainable artificial intelligence–based clinical decision support systems (XAI-CDSSs) into routine mental health screening and care could enhance early detection, risk stratification, and individualised intervention planning [59].

Our correspondence analysis results also revealed a high prevalence of stress and anxiety and a risk for depression among the 15–20 and 21–30 age groups, potentially sharing similarities regarding such mental health problems. The 31–40, 41–50, and 51–60 age groups exhibited a close association with social support and coping flexibility, which might make them more resilient to psychological problems. This result has been attributed to the fact that these age groups generally comprise married individuals with children, those employed in income-generating jobs, those lacking health issues that would significantly impact their social relationships or psychological well-being, and those who have not yet experienced the loss of a spouse or close relative. Lazarus and Folkman also reported that coping mechanisms change with age, that middle-aged individuals can develop more effective coping strategies, and that having more effective social support mechanisms plays a major role in this [60]. Depression was the most relevant factor for the 61–75 age group when compared with all other factors. We believe that, because the elderly population in our study were smartphone users, they could access accurate and reliable information about the pandemic, learn and implement appropriate precautions, and thus, create an environment in which they could protect themselves adequately. This, in turn, might have led to them exhibiting fewer symptoms of stress and anxiety during this period [61]. Moreover, their longevity and maturity can enable them to view life more positively, and the emotional and psychological resilience acquired through their life experiences enables more effective management of anxiety and stress [62]. It was concluded that their influence by depression was not solely due to the pandemic, but rather attributed to a lack of social support and coping flexibility, as well as the high likelihood of chronic diseases affecting their functioning and having experienced a loss of relatives.

Limitations: This study reveals the interplay between age and psychological factors that occurred during the COVID-19 pandemic and provides guidance for mental health support policies in the context of pandemics. However, it still has some limitations. The cross-sectional design of the study limits the ability to establish causality, for which longitudinal studies are required. As the results are based on individuals’ own statements, there is a possibility of recall bias. The study has addressed social support and coping flexibility as factors that increase mental resilience, but other factors that have positive effects on mental health (e.g., physical activity) should not be overlooked [11]. Furthermore, as the study was conducted in only one city, it cannot be generalised to the wider country or global populations. Cultural, economic, and healthcare factors specific to this region may not translate toother contexts. Nevertheless, we believe that the broad and meaningful insights obtained from this research provide valuable guidance for the development and implementation of public health, policy, and technology-based interventions during crises such as pandemics, serving as informative rather than generalizable evidence. As the data were based on online scale assessments, no clinical interviews were conducted, and no diagnoses were made for anxiety and depression. Another limitation is that none of the participants had a clinical diagnosis or assessment prior to the pandemic, making it difficult to determine how much of the current findings can be attributed to the pandemic alone. Due to social restrictions, a random sampling method could not be applied, so a convenience method was used instead. Therefore, the participants in this study might have experienced more severe COVID-related problems or stress and wished to report this; thus, the results cannot be generalised. It has been demonstrated that during the pandemic, elderly individuals may have exhibited different and increasingly severe psychological problems in every aspect of their lives [12]. However, as their rates of literacy and smartphone usage are lower, the number of individuals over 60 years of age in the study remained limited and they were examined as a single group, thus failing to reach a sufficient number to identify differences within this group. The correspondence analysis, although visually informative, may oversimplify complex relationships by dichotomizing continuous variables based on mean splits. This methodological choice can lead to a loss of nuanced information and the creation of artificial distinctions between age groups. Nevertheless, this visualisation enhances interpretability by highlighting patterns that might otherwise remain obscured. Despite its limitations, we consider this approach valuable for informing policy development, guiding technology-supported and community-based interventions, and facilitating the monitoring of outcomes over time. As additional questions could prolong the survey and have the potential to reduce participation rates, the questions about potential problems and fears related to COVID-19 have been phrased as single sentences instead of using standardised instruments. In the absence of a specific measurement tool, different participants might have interpreted them differently.

## 5. Conclusions

This study shows that mental health is highly affected in pandemics. It has been concluded that, even if the cause is the same, different mental health issues can arise at different stages of life. Therefore, mental health issues, as well as resilience factors such as social support and coping flexibility, should be tailored to age-specific vulnerabilities, prioritising youth for stress/anxiety interventions and older adults for depression support. Regular monitoring of the community in terms of a pandemic’s effects and the implementation of appropriate programmes to provide emotional, mental, and social support should be the primary objectives in future pandemics, with priority given to women, young people, the elderly, people living alone, people experiencing COVID-19-related fears and concerns intensely, and those experiencing problems with control, sleep, and concentration. Early identification of affected groups is essential for the development and effective implementation of interventional strategies. Furthermore, it is of great importance that protective and preventive measures are designed and implemented separately and specifically for each age group.

Taken together, the COVID-19 pandemic placed a substantial psychological burden on communities, affecting disproportionately younger adults. These results highlight the need for age-tailored, resilience-focused, technology-supported, community-based mental health programmes to enhance population well-being during future public health emergencies.

## Figures and Tables

**Table 1:** Distribution of sociodemographic and COVID-19-related descriptive features of the participants.

Sociodemographic Features	n	%
Gender		
Female	986	58.0
Male	713	42.0
Age		
15–20	215	12.7
21–30	612	36.0
31–40	368	21.7
41–50	282	16.6
51–60	143	8.4
61–75	79	4.6
Marital Status		
Single	840	49.4
Married	793	46.7
Divorcee/widow	66	3.9
Education		
High school or less	349	20.5
Bachelor or more	1350	79.5
Employment		
No	206	12.1
Yes	892	52.5
Student	516	30.4
Retired	85	5.0
Chronic Disease		
No	1430	84.2
Yes	269	15.8
Chronic Patient at Home		
No	1131	66.6
Yes	568	33.4
Easy Access to Personal Protective Equipment		
No	243	14.3
Yes	1456	85.7
Having Been Quarantined		
No	982	57.8
Yes	717	42.2
Need for Psychologic Support		
No	1257	74.0
Yes	442	26.0
Anxiety Symptoms		
No	1282	75.5
Yes	417	24.5
Depressive Symptoms		
No	983	57.9
Yes	716	42.1

**Table 2:** Correlation coefficients and mean values of scale scores, COVID-19-related problems, and fears.

	CFS	PSS	MPSSS	HADS-A	HADS-D	Mean ± SD (Min–Max)
Age	0.033	−0.229 **	0.136 **	−0.113 **	−0.085 **	34.1 ± 13.1 (15–75)
No. of household members	−0.095 **	0.126 **	−0.181 **	0.096 **	0.087 **	4.3 ± 1.9 (1–16)
Feeling lonely	−0.214 **	0.428 **	−0.249 **	0.462 **	0.443 **	2.5 ± 1.2 (1–5)
Feeling distant from others	−0.145 **	0.362 **	−0.154 **	0.404 **	0.373 **	2.8 ± 1.2 (1–5)
Feeling of losing control over life	−0.210 **	0.482 **	−0.207 **	0.504 **	0.462 **	2.5 ± 1.3 (1–5)
Feeling ostracised	−0.223 **	0.357 **	−0.182 **	0.449 **	0.417 **	1.9 ± 1.2 (1–5)
Sleep problems	−0.177 **	0.388 **	−0.167 **	0.470 **	0.425 **	2.5 ± 1.3 (1–5)
Concentration problems	−0.198 **	0.447 **	−0.188 **	0.510 **	0.462 **	2.5 ± 1.3 (1–5)
Fear of contracting COVID-19	−0.098 **	0.244 **	0.048 *	0.380 **	0.229 **	5.7 ± 2.9 (0–10)
Fear of dying from COVID-19	−0.073 **	0.242 **	0.057 *	0.380 **	0.216 **	5.3 ± 3.3 (0–10)
CFS		−0.412 **	0.362 **	−0.356 **	−0.398 **	20.4 ± 5.4 (2–30)
PSS			−0.362 **	0.619 **	0.599 **	27.4 ± 7.6 (0–56)
MPSSS				−0.238 **	−0.331 **	60.6 ± 17.4 (12–84)
HADS-A					0.699 **	8.5 ± 4.5 (0–21)
HADS-D						7.8 ± 4.1 (0–21)

* *p* < 0.05, ** *p* < 0.01. SD: standard deviation; CFS: Coping Flexibility Scale; PSS: Perceived Stress Scale; MPSSS: Multidimensional Perceived Social Support Scale; HADS-A: Hospital Anxiety Depression Scale—Anxiety Section; HADS-D: Hospital Anxiety Depression Scale—Depression Section.

**Table 3:** Comparison of scale scores with sociodemographic and COVID-19-related descriptive characteristics.

Sociodemographic Variables	CFS Mean ± SD	PSS Mean ± SD	MPSSS Mean ± SD	HADS-A Mean ± SD	HADS-D Mean ± SD
Gender					
Female	20.3 ± 5.5	28.8 ± 7.4	61.1 ± 17.7	9.3 ± 4.5	8.1 ± 4.2
Male	20.5 ± 5.1	25.5 ± 7.6	59.9 ± 17.0	7.3 ± 4.2	7.2 ± 4.0
t	−0.667	8.868 **	1.450	9.263 **	3.604 **
ES ^†^	−0.033	0.436	0.071	0.455	0.177
Age					
15–20 ①	19.8 ± 5.5	29.1 ± 8.3	54.4 ± 18.0	9.2 ± 4.4	8.4 ± 4.2
21–30 ②	20.2 ± 5.4	29.1 ± 7.3	59.1 ± 17.2	8.8 ± 4.5	8.0 ± 4.1
31–40 ③	20.7 ± 5.2	26.8 ± 6.8	64.0 ± 16.5	8.4 ± 4.4	7.6 ± 4.1
41–50 ④	20.6 ± 5.3	25.4 ± 8.3	62.2 ± 17.2	8.0 ± 4.6	7.2 ± 4.2
51–60 ⑤	21.4 ± 5.5	23.9 ± 7.3	64.5 ± 17.8	7.5 ± 4.3	6.8 ± 3.9
61–75 ⑥	19.0 ± 5.3	25.5 ± 5.1	60.6 ± 16.2	7.9 ± 3.6	8.2 ± 4.0
F	2.997 *	20.819 **	11.337 **	4.288 **	4.336 **
ES ^‡^	0.009	0.058	0.032	0.013	0.013
Post hoc test	⑤ > ⑥	①, ② > ③, ④, ⑤, ⑥; ③ > ⑤	① < ②, ③, ④, ⑤; ② < ③, ⑤	① > ④, ⑤; ② > ⑤	① > ④, ⑤; ② > ⑤
Marital Status					
Single ①	20.1 ± 5.5	28.9 ± 7.8	57.4 ± 17.6	8.9 ± 4.5	8.1 ± 4.1
Married ②	20.5 ± 5.3	25.9 ± 7.4	64.1 ± 16.7	8.1 ± 4.4	7.4 ± 4.1
Divorced/widow ③	21.8 ± 5.3	25.8 ± 7.6	59.8 ± 15.9	7.9 ± 4.0	7.5 ± 4.5
F	3.488 *	35.120 **	31.669 **	6.572 **	5.380 **
ES ^‡^	0.004	0.040	0.036	0.008	0.006
Post hoc test	③ > ①	① > ②, ③	② > ①	① > ②	① > ②
Education					
High school or less	19.6 ± 5.26	27.5 ± 6.7	58.6 ± 18.0	8.9 ± 4.3	8.2 ± 4.0
Bachelor or more	20.6 ± 5.4	27.4 ± 7.9	61.1 ± 17.2	8.4 ± 4.5	7.6 ± 4.2
t	−3.147 **	0.280	−2.348 *	2.017 *	2.358 *
ES ^†^	−0.189	0.017	−0.142	0.121	0.142
Employment					
No ①	20.1 ± 4.9	27.5 ± 6.5	62.0 ± 18.1	8.9 ± 4.5	7.9 ± 3.7
Yes ②	20.8 ± 5.4	26.6 ± 7.8	63.1 ± 16.9	8.2 ± 4.5	7.3 ± 4.2
Student ③	19.7 ± 5.4	29.4 ± 7.8	55.2 ± 17.0	9.1 ± 4.4	8.4 ± 4.1
Retired ④	20.1 ± 5.8	23.8 ± 6.2	63.9 ± 16.2	7.3 ± 3.8	7.7 ± 4.2
F	4.991 **	24.968 **	25.462 **	7.229 **	7.506 **
ES ^‡^	0.009	0.038	0.043	0.013	0.013
Post hoc test	② > ③	③ > ①, ②, ④; ① > ④	③ < ①, ②, ④	① > ④; ③ > ②, ④	③ > ②
Chronic Disease					
No	20.5 ± 5.3	27.4 ± 7.7	60.7 ± 17.3	8.4 ± 4.5	7.7 ± 4.2
Yes	19.9 ± 5.6	27.2 ± 7.5	60.2 ± 18.1	8.8 ± 4.3	7.8 ± 4.0
t	1.626	0.549	0.440	−1.159	−0.304
ES ^†^	0.108	0.036	0.029	−0.077	−0.020
Chronic Patient at Home					
No	20.6 ± 5.3	26.6 ± 7.9	61.6 ± 17.1	8.0 ± 4.5	7.4 ± 4.1
Yes	19.8 ± 5.4	29.0 ± 6.9	58.6 ± 17.9	9.5 ± 4.3	8.5 ± 4.1
t	2.982 **	−6.415 **	3.427 **	−6.444 **	−5.131 **
ES ^†^	0.153	−0.316	0.176	−0.331	−0.264
Easy Access to PPE					
No	19.0 ± 5.1	28.8 ± 6.5	55.8 ± 16.2	9.6 ± 4.2	9.1 ± 4.0
Yes	20.6 ± 5.4	27.2 ± 7.8	61.4 ± 17.5	8.3 ± 4.5	7.5 ± 4.1
t	−4.255 **	2.654 **	−4.960 **	4.235 **	5.415 **
ES ^†^	−0.295	0.162	−0.325	0.293	0.375
Having Been Quarantined					
No	20.5 ± 5.3	26.6 ± 7.3	61.7 ± 17.0	8.2 ± 4.4	7.5 ± 4.0
Yes	20.1 ± 5.5	28.5 ± 8.0	59.0 ± 18.0	8.9 ± 4.6	8.1 ± 4.3
t	1.545	−5.181 **	3.125 **	−3.305 **	−3.038 **
ES ^†^	0.076	−0.255	0.155	−0.162	−0.149
Need for Psychological Support					
No	20.8 ± 5.2	25.9 ± 7.4	62.2 ± 16.9	7.3 ± 4.1	6.8 ± 3.8
Yes	19.2 ± 5.6	31.7 ± 6.7	56.1 ± 18.1	11.8 ± 3.9	10.4 ± 3.9
t	5.303 **	−14.737 **	6.361 **	−19.917 **	−16.874 **
ES ^†^	0.293	−0.815	0.352	−1.101	−0.933
Anxiety Symptoms					
No	21.2 ± 5.1	25.5 ± 7.1	62.3 ± 16.9	-	6.6 ± 3.6
Yes	17.8 ± 5.4	33.1 ± 6.4	55.3 ± 18.0	-	11.4 ± 3.6
t	11.701 **	−20.425 **	7.287 **	-	−23.941 **
ES ^†^	0.660	−1.090	0.411	-	−1.350
Depressive Symptoms					
No	21.8 ± 5.0	24.4 ± 7.2	64.6 ± 16.3	6.4 ± 3.7	-
Yes	18.4 ± 5.3	31.5 ± 6.1	55.1 ± 17.5	11.4 ± 3.71	-
t	13.516 **	−21.238 **	11.576 **	−27.277 **	-
ES ^†^	0.664	−1.043	0.569	−1.340	-

* *p* < 0.05; ** *p* < 0.01. The numbers next to the age groups have been used to display that group more concisely and clearly in post-hoc comparisons. PPE: personal protective equipment; SD: standard deviation; CFS: Coping Flexibility Scale; PSS: Perceived Stress Scale; MPSSS: Multidimensional Perceived Social Support Scale; HADS-A: Hospital Anxiety Depression Scale—Anxiety Section; HADS-D: Hospital Anxiety Depression Scale—Depression Section; ES: effect size; ^†^: Cohen’s d; ^‡^: eta squared (η^2^).

**Table 4:** Linear regression models of age, COVID-19-related problems and fears, and scale scores for anxiety and depressive symptoms.

Model Variables	Anxiety Symptoms			Depressive Symptoms		
	B (SE)	%95 CI for B	β	B (SE)	%95 CI for B	β
Age	−0.004 (0.005)	−0.015; 0.006	−0.013	0.014 ** (0.005)	0.004; 0.025	0.045
No. of household members	0.092 * (0.036)	0.021; 0.162	0.040	−0.023 (0.036)	−0.094; 0.048	−0.011
Feeling lonely	0.048 (0.083)	−0.015; 0.210	0.013	0.119 (0.083)	−0.044; 0.281	0.035
Feeling distant from others	−0.008 (0.075)	−0.155; 0.140	−0.002	0.042 (0.075)	−0.106; 0.190	0.013
Feeling of losing control over life	0.156 * (0.075)	0.009; 0.303	0.045	0.094 (0.075)	−0.052; 0.241	0.030
Feeling ostracised	0.273 ** (0.075)	0.126; 0.421	0.073	0.119 (0.075)	−0.029; 0.267	0.034
Sleep problems	0.201 ** (0.071)	0.063; 0.340	0.059	0.116 (0.071)	−0.023; 0.254	0.037
Concentration problems	0.282 ** (0.078)	0.128; 0.435	0.079	0.149 (0.079)	−0.005; 0.303	0.045
Fear of contracting COVID-19	0.149 ** (0.033)	0.083; 0.214	0.097	−0.021 (0.033)	−0.087; 0.044	−0.015
Fear of dying from COVID-19	0.171 ** (0.029)	0.114; 0.228	0.126	−0.048 (0.029)	−0.105; 0.010	−0.038
Coping flexibility	−0.042 ** (0.014)	−0.070; −0.014	−0.050	−0.071 ** (0.014)	−0.099; −0.043	−0.092
Perceived stress	0.115 ** (0.012)	0.091; 0.139	0.197	0.101 ** (0.012)	0.077; 0.125	0.187
Perceived social support	0.008 (0.004)	−0.001; 0.016	0.031	−0.021 ** (0.004)	−0.029; −0.012	−0.087
Anxiety symptoms	-	-	-	0.430 ** (0.022)	0.387; 0.474	0.464
Depressive symptoms	0.431 ** (0.022)	0.387; 0.474	0.399	-	-	-
Model [F(df); p]	205.025(14); <0.001			159.412(14); <0.001		
Adjusted R^2^	0.627			0.566		

* *p* < 0.05; ** *p* < 0.01. B: unstandardized coefficients; SE: standard error; CI: confidence interval; β: standardised coefficients.

## Data Availability

The raw data supporting the conclusions of this article will be made available by the authors on request.
